# Body Composition and Phase Angle: How to Improve Nutritional Evaluation in Juvenile Dermatomyositis Patients

**DOI:** 10.3390/nu15133057

**Published:** 2023-07-07

**Authors:** Camila Pugliese, Artur Figueiredo Delgado, Katia Tomie Kozu, Lucia Maria de Arruda Campos, Nadia Emi Aikawa, Clovis Artur Silva, Adriana Maluf Elias

**Affiliations:** 1 Nutrition Unit, Children and Adolescent’s Institute, Hospital das Clínicas HCFMUSP, Faculdade de Medicina, Universidade de São Paulo, Av. Dr. Enéas Carvalho de Aguiar, 647-Cerqueira César, São Paulo 05403-000, SP, Brazil; 2 Intensive Care Unit, Children and Adolescent’s Institute, Hospital das Clínicas HCFMUSP, Faculdade de Medicina, Universidade de São Paulo, Av. Dr. Enéas Carvalho de Aguiar, 647-Cerqueira César, São Paulo 05403-000, SP, Brazil; 3 Rheumatology Unit, Children and Adolescent’s Institute, Hospital das Clínicas HCFMUSP, Faculdade de Medicina, Universidade de São Paulo, Av. Dr. Enéas Carvalho de Aguiar, 647-Cerqueira César, São Paulo 05403-000, SP, Brazil

**Keywords:** bioelectrical impedance, nutritional assessment, adolescents

## Abstract

(1) Background: This study aimed to assess body composition (BC) using bioelectrical impedance and food intake in juvenile dermatomyositis (JDM) patients. Associations between BC and physical activity, disease activity/cumulative damage and health-related quality of life parameters were also evaluated; (2) Methods: This was a cross-sectional study with 30 consecutive JDM patients (18 female and 12 male) and 24 healthy volunteers (14 female and 10 male) of both sexes followed at our pediatric rheumatology unit. The gathering of anthropometric and dietary data, and the performance of physical activity and bioelectrical impedance were undertaken in face-to-face meetings and through questionnaires. Clinical and therapeutic data were collected from medical records according to information from routine medical appointments; (3) Results: The frequency of high/very high body fat was significantly higher in controls compared with JDM patients (66.7% vs. 91.7%; *p* = 0.046). The median phase angle was significantly lower in patients compared with controls (5.2 ± 1.3 vs. 6.1 ± 1.0; *p* = 0.016). Body fat and lean mass were positively correlated with disease duration (r_s_ = +0.629, *p* < 0.001 and r_s_ = +0.716, *p* < 0.001, respectively) and phase angle (PhA) (r_s_ = +0.400, *p* = 0.029 and r_s_ = +0.619, *p* < 0.001, respectively). JDM patients with PhA ≥ 5.5 presented higher lean mass when compared with patients with PhA < 5.5 (*p* = 0.001); (4) Conclusions: Bioelectrical impedance can be a useful auxiliary exam in the medical and nutritional follow-up of JDM patients, because it seems to impact functional ability. These findings may assist professionals when advising JDM patients about the importance of physical activity and healthy eating in the preservation of lean mass.

## 1. Introduction

Juvenile dermatomyositis (JDM) is a chronic systemic autoimmune muscle disorder that begins in children and adolescents under 18 years of age and is the most common type of juvenile idiopathic inflammatory myopathy. It is characterized by proximal weakness, mainly affecting the skin and proximal muscles [1]. The incidence is of around two to three cases/million children per year in the general population [2]. Studies have shown that JDM patients may be at increased risk of obesity [3] and of dyslipidemia [4,5], premature atherosclerosis [6] and low lean mass [7]. These findings are thought to be due to both disease-specific factors and a host of traditional risk factors that can be related to irregular physical activity [8] and bad eating habits [9].

Bioelectrical impedance analysis (BIA) is a validated method frequently used to assess body composition. This is an easy, safe and inexpensive method to use in clinical practice [10,11]. Body composition should be measured independently of a person’s clinical condition and as part of their nutritional status assessment as low lean mass and high body fat can be related to the worsening of chronic diseases [11]. Phase angle (PhA) is another important screening tool, one which integrates the assessment by BIA, and is used to identify patients at risk of the deterioration of their nutritional status, their functionality and their prognosis of mortality [12]. Beyond that, PhA is the most established impedance parameter related to clinical prognosis in many diseases and is associated with changes in cellular membrane integrity and alterations in fluid balance [13]. Lower PhA values represent low reactance (Xc) and high resistance (R), which can be associated with selective membrane permeability, cell death and the worsening of disease. In contrast, higher PhA values represent high Xc and low R, which can be associated with a greater amount of intact cell membranes and body cell mass and therefore with an adequate state of health [14]. PhA reference values are currently lacking for children and adolescents [13,14]. This parameter therefore indicates changes in body composition and cell membrane function in the health status of individuals [14]. Indeed, the European Society for Clinical Nutrition and Metabolism (ESPEN) strongly recommends the assessment of PhA, which is a nutritional measure and a reliable prognostic marker [11,15].

Clinical research reports have demonstrated a high intake of saturated fat and low intake of micronutrients in different groups of children and adolescents, including patients with autoimmune rheumatic diseases. However, this sample of young patients contain a small representation of JDM (n = 20) [3,16]. Strong evidence suggests that poor food consumption, lacking in fiber, vitamins and minerals and with excessive fat and sugar, can contribute to the promotion of adipogenesis, to the increased risk of obesity and to other cardiometabolic and chronic diseases [16]. However, to the best of our knowledge there is no study that has simultaneously evaluated body composition, phase angle and food consumption in JDM patients.

Pediatric patients with rheumatic diseases tend to be hypoactive. Increasing levels of physical activity and reducing sedentary behavior have been shown to improve mechanical, physical and biochemical processes, as well as quality of life [17]. The hypothesis is that the duration of the disease may be related to the prognosis (phase angle) and other clinical and demographic parameters. Therefore, the objective of this study was to perform a nutritional assessment in JDM patients and healthy controls in order to determine dietary intake and body composition and the potential influence of demographic data, physical activity, disease activity, treatment, and health-related quality of life parameters.

## 2. Materials and Methods

What were the instruments used for measuring height, weight, skinfolds and bioelectrical impedance?

### 2.1. Participants

A cross-section study was performed from November 2017 to April 2021, and included 31 JDM patients followed at the pediatric rheumatology unit of our university hospital. Inclusion criteria were the diagnosis of JDM according to the following criteria developed by Bohan and Peter [18]. 1. Symmetrical weakness, usually progressive, of the limb-girdle muscles with or without dysphagia and respiratory muscle weakness. 2. Muscle biopsy evidence of myositis necrosis of type I and type II muscle fibers; phagocytosis, degeneration, and regeneration of myofibers with variation in myofiber size; and the presence of endomysial, perimysial, perivascular, or interstitial mononuclear cells. 3. Elevation of serum levels of muscle-associated enzymes (creatinine kinase, lactate dehydrogenase, transaminases, aldolase). 4. Electromyography triad of myopathy: short, small, low-amplitude polyphasic motor unit potentials; fibrillation potentials, even at rest; and high-frequency repetitive discharges. 5. Characteristic rashes of dermatomyositis.

Exclusion criteria were pregnancy and the use of a pacemaker, due to the bioelectrical impedance examination performed. Patients who chose not to participate in the study or who had another associated disease were excluded. All JDM patients were evaluated by the pediatric rheumatologist and the pediatric nutritionist that had performed the nutritional assessment and the bioelectrical impedance examination. The control group consisted of 25 healthy children and adolescents who consented to participate in the study, were randomly selected, and were either known or unknown to the patients. One JDM patient and one healthy subject refused to participate due to lack of time. Therefore, the final group was composed of 30 JDM patients and 24 healthy controls.

Demographic data included current age, sex, weight (kilograms), height (meters) and disease duration. Weight was measured on a digital scale with a duly calibrated precision scale before each weighing (Welmy RI W 200^®^, 100 g precision scale, Welmy, São Paulo, Brazil). Height was obtained using an aluminum stadiometer fixed to the wall with a precision scale of 0.1 cm. Body mass index (BMI) was defined by the formula: weight (kilograms) divided by square of height (meters). To characterize the nutritional status, anthropometric data were assessed using the Z-score of BMI for age and Z-score of height for age. Clinical and treatment data were obtained from institutional medical records. The World Health Organization (WHO) Anthro Plus software (WHO AnthroPlus, GE, Switzerland) was used to calculate the Z-scores. Overweight was defined as Z-score >+1 and ≤+2 standard deviation (SD) for BMI, obesity as Z-scores >+2 and ≤+3 SD for BMI and severe obesity as Z-scores >+3 SD for BMI [19]. Parents and participants were asked to rate their child’s usual level of physical activity on a five-point scale, based on the last 6 months: “daily”, “several times a week”, “once a week”, “more seldom” and “never”. This is a simple and often-used method to provide information on activity levels of the studied population [20]. This study was approved by the Institutional Ethics Committee of our university hospital (CAAE: 96002618.4.0000.0068; Number: 3.402.544) and appropriate, written, and informed consent was obtained from all participants and their legal guardians.

### 2.2. Dietary Assessment

Dietary intake was assessed using 2 or 3 24 h dietary recalls for each patient—including one for weekends and one or two for the days of the week—which is an instrument based on a structured interview intended to capture detailed information about all foods and beverages and their amounts consumed by the respondent during the 24 h prior to the interview. To calculate energy intake (kilocalories), macronutrients (carbohydrates, proteins, total fats, saturated fats and fiber) and micronutrients, expressed in grams per day, as well as the percentage of energy provided by each macronutrient (acceptable macronutrient distribution ranges (AMDR)) and antioxidant micronutrients, the online Avanutri Nutrition Software was used. The values were classified according to the dietary reference intakes (DRIs) for the following parameters: recommended dietary allowance (RDA) or adequate intake (AI)–AI being used when RDA could not be determined–and tolerable upper intake level (UL) of macro and micronutrients [21]. The Food and Agriculture Organization/WHO was the reference we used to evaluate saturated fats [22]. Fiber intake was assessed using the references in the American Health Foundation’s recommendation, according to which the minimum daily fiber intake (in grams) is equal to the child’s age (in years) plus five [23]. All these data were collected, analyzed and calculated by an experienced nutritionist, blinded to disease activity and health-related quality of life parameters.

### 2.3. Body Composition Evaluation

Body composition (fat and lean mass and their percentages) was evaluated by bioelectrical impedance, using the tetrapolar model Biodynamics 450 (Body Composition Analysis Biodynamics Corporation, Seattle, WA, USA) with a standard current of 800 µA and frequency of 50 kHz. Adhesive electrodes were placed at anatomical sites on the dorsal surfaces of the hand, wrist, ankle, and foot, following the preparation and technique inherent to the exam and according to the manufacturer’s instructions. This consists of a non-invasive exam that most accurately assesses body composition. Using body impedance and total body water as input variables, it is possible to predict the % of body fat, fat mass and lean mass. BIA is already validated for use in children and adolescents, but not the equations contained within it, so these equations are considered less accurate for younger children. In addition to the prediction of body fat by BIA, Slaughter and Deurenberg equations—which are stratified by age and sex and have been validated for use on children and adolescents—were used to assess the % of body fat of the participants. The PhA is an index of the ratio between extracellular and intracellular water, body cell mass, and cellular integrity and was obtained directly by BIA analysis, with no needed equations. The value can be obtained through the relationship between direct measurements of R and Xc, being calculated directly by the equation Xc/R × 180°/¶. The median PhA in JDM patients (5.5) was used as the cut-off point to divide the group.

Arm and waist circumference were measured with flexible tape and skinfold measurements (bicipital, tricipital, subscapular and suprailiac skin fold) were also taken by adipometer (Lange^®^ skinfold calipter, Beta Technology Inc., Cambridge, MA, USA) and classified to measure fat mass [24,25]. Slaughter and Deurenberg equations stratified by sex, ethnicity (Afro-Latin American and Caucasian) and according to the sum of triceps and subscapular skinfold were used to estimate the percentage of body fat [26,27].

Body fat percentage was classified using the values proposed by Lohman et al. (1992) [28]: very low (≤6%), low (between 7 and 12%), normal (between 13 and 18%), high (between 19 and 32%) and very high (≥32%).

Waist circumference (WC) was measured with the child standing, using an inelastic tape graduated in millimeters in the space corresponding to the smallest circumference between the iliac crest and the costal margin at the moment of maximal exhalation. Subjects whose WC values were equal to or greater than the 80th percentile were considered to have central obesity [29]. The waist-to-height ratio was calculated as an anthropometric marker of central adiposity and health risk for adults, considering the cut-off point of 0.50 for all age groups and both genders [30,31].

Clinical lipodystrophy was defined as the presence of lipoatrophy and/or lipohypertrophy [32].

### 2.4. JDM Clinical and Treatment Assessments

Disease activity and muscle strength were systematically measured by the Disease Activity Score (DAS), the Childhood Myositis Assessment Scale (CMAS), and the Manual Muscle Test (MMT) [33,34,35], in addition to the presence of typical signs and symptoms of active JDM, including the presence of rashes, increasing calcinosis lesions, weakness, and elevated muscle enzymes. The DAS consists of 19 items, resulting in a total score of 0 to 20. The parameters of disease activity used were a Disease Activity Score (DAS) ≥ 3, a Childhood Myositis Assessment Scale (CMAS) score < 48 and a Manual Muscle Strength Test (MMT) score < 78. Myositis damage index extent (MDI-extent) > 0 and myositis damage index severity (MDI-severity) > 0 were used as parameters of cumulative damage, as well as the presence of calcinosis [33,34,35].

JDM scores included the validated Brazilian version of the Childhood Health Assessment Questionnaire (CHAQ), wherein results > 0 indicate functional disability [36]. The generic instrument Pediatric Quality of Life Inventory 4.0 (PedsQL 4.0), validated for use in Brazilian Portuguese, was also performed using different versions for the following three age groups: 5–7, 8–12 and 13–18 years old. Two instruments were used in the present study: PedsQl 4.0 child self-report <78 and PedsQl 4.0 parents report <78 were indicative of compromised functional abilities and health-related quality of life deterioration parameters, respectively [37,38].

Duration of disease, cumulative dose of glucocorticoids (GC), and intravenous pulse therapy use were obtained from medical records. Data concerning the use and current dosage of prednisone, methylprednisolone, methotrexate, immunosuppressive drugs (cyclosporine, azathioprine, mycophenolate mofetil) and biological agents (adalimumab, etanercept, rituximab) were also assessed.

### 2.5. Statistical Analysis

Statistical analysis was performed using MedCalc Statistical Software version 17.8.6 (MedCalc Software bvba, Ostend, Belgium). Data are presented in median (range) or mean ± standard deviation (SD) for continuous variables according to abnormal or normal distribution (Shapiro–Wilk test), respectively. Age, weight, height, body fat (g and %), lean mass (g and %) and PhA were considered variables with normal distribution. Data are presented in number (percentages) for categorical variables. For continuous variables, data were compared using Mann–Whitney for non-normal distribution or Student’s *t*-test for normal distribution to evaluate differences between JDM patients and healthy controls, and patients with PhA ≥ 5.5 and with PhA < 5.5 according to body fat percentage, food intake, Health Related Quality of Life (HRQL) scores, clinical parameters, and therapeutic data. Analysis of variance (ANOVA) was used to compare body fat percentage measurements by bioelectrical impedance and Slaughter and Deurenberg equations. For categorial variables, differences were assessed by the Fisher’s exact test or Pearson’s chi-square test. In JDM patients Pearson’s correlation coefficient was used to investigate the relationship between PhA and parameters of disease activity, cumulative damage, HRQL and treatment. *p* values less than 0.05 (5%) were considered significant. Effect sizes were calculated according to a previous description [39]. Cohen’s *d* represents effect sizes: small effect: d ≥ 0.2; average effect: d ≥ 0.5; big effect: d ≥ 0.8 and below 0.2 is trivial.

## 3. Results

### 3.1. JDM patients vs. Healthy Controls

Demographic, anthropometric, body composition, clinical data and treatments, and food intake in JDM patients and healthy controls are shown in Table 1. The current age was similar in JDM patients and healthy controls (11.8 ± 4.0 vs. 10.7 ± 3.1 years; *p* = 0.249), as well as sex distribution (18 female:12 male vs.14 female:10 male; *p* = 0.924). Median age at JDM diagnosis was 6.3 (1.4–11.4) years and median disease duration was 3.3 (0.21–16.6) years. Disease activity was observed in 10/30 (33.3%) and 90% used corticosteroids at least once during treatment. The mean cumulative dose of corticosteroids was 12,229.0 ± 10,100.0 mg.

Of a total of nearly 37% of overweight JDM patients, 5 were obese (16.7%), a similar proportion to that found in healthy controls (3 patients; 12.5%) (*p* = 0.720). There was no statistically significant difference between the groups regarding waist circumference/height ratio, but approximately 37% of JDM patients were at cardiovascular risk and 31.5% of central adiposity was found in the total sample. The median phase angle was significantly lower in JDM patients compared with controls (5.2 ± 1.3 vs. 6.1 ± 1.0; *p* = 0.016). Although there were no differences concerning macronutrients between groups, JDM patients had significantly higher fiber consumption than healthy individuals (17.3 (2.6–40.9 g) vs. 11.9 (4.6–25.1 g); *p* < 0.0001) (Table 1). Both JDM patient and control groups presented a higher percentage of inadequacy in fiber consumption (53.3% vs. 70.8%) (Table 2).

There was a statistically significant difference in the percentage of fat mass between JDM patients compared with controls concerning bioelectrical impedance and Deurenberg equation (25.3 ± 6.7% vs. 21.3 ± 4.8%; *p* < 0.05), and Slaughter and Deurenberg equations (26.9 ± 8.1% vs. 21.3 ± 4.8%; *p* < 0.05). No difference was found between the percentage of fat mass between JDM patients compared with controls concerning bioelectrical impedance and Slaughter equation (25.3 ± 6.7% vs. 26.9 ± 8.1%; *p* = 0.914).

Table 2 shows the adequacy of dietary intake between JDM patients and healthy controls based on the recommendations of the Dietary Reference Intake (DRI). More than 33% of healthy controls and almost 17% of JDM patients were above acceptable macronutrient distribution ranges (AMDR) of total fat. It was observed that the adequacy of selenium intake was significantly lower in JDM patients compared with healthy controls (30% vs. 62.5%; *p* = 0.027).

### 3.2. JDM Patients

Comparing JDM patients with PhA < 5.5 and PhA ≥ 5.5, it is observed that current age was significantly higher in patients with PhA ≥ 5.5 (14.1 ± 3.9 vs. 9.8 ± 3.1 years; *p* = 0.003) and disease duration was significantly lower in JDM patients with PhA < 5.5 (2.2 (0.2–8.6) vs. 7.5 (2.7–16.6) years; *p* < 0.001) (Table 3).

Of note, in JDM patients, a positive correlation was identified between phase angle and disease duration (r_s_ = +0.648, *p* < 0.001) and negative correlations were identified between phase angle and DAS total score (r_s_ = −0.515, *p* = 0.004), DAS muscle (r_s_ = −0.598, *p* < 0.001) and DAS skin (r_s_ =−0.381, *p* = 0.038), which was not observed for HRQL scores. There was no correlation between phase angle and macro or micronutrient intake.

## 4. Discussion

The aim of the study was to perform a nutritional assessment of JDM patients and healthy controls, including body composition and dietary intake as well as their relation to physical activity, demographic data, disease activity, treatment, and health-related quality-of-life parameters.

Although there were differences in the Z-score height/age between patients and healthy controls, only one (3.3%) JDM patient was identified with short stature. Several studies have been published showing compromised stature in pediatric patients with rheumatological diseases, secondary to disease duration and prolonged use of drugs, with prevalence ranges from 10% to 40% [40,41].

Generally, patients have a worse nutritional status as a result of disease itself, inflammatory processes, GC and limited physical activity [6,7,8]. Although we observed that healthy controls presented higher body fat percentages compared with JDM patients, the percentages of those overweight and obese were similar in both groups. Increased consumption of ultra-processed foods can result in weight gain and may have a significantly negative effect on the body composition, and physical and nutritional health of children and adolescents [42].

RDA is the average daily dietary intake level sufficient to meet the nutrient requirements of nearly all (97–98 percent) healthy individuals in a group [21]. Concerning macronutrients, the majority of both groups ingested within the RDA recommendations. It was found that 71% of JDM patients had an inadequacy of fiber intake; in addition, more than half of the controls did not meet fiber recommendations. In both evaluated groups, this fact was related to a low intake of fruits, vegetables and whole foods, as well as to the high consumption of snacks, sweetened drinks, stuffed cookies and fast food. The high intake of ultra-processed foods can also be related to the high content of saturated fat in their usual diet.

Few JDM patients and healthy controls reported using vitamin and/or mineral supplements. Micronutrient supplementation for JDM patients, especially calcium, should be recommended, because calcium intake from food is less than ideal and there is bone involvement in the disease. The antioxidant selenium showed a high prevalence of inadequate intake in the JDM patients’ group—the diet of both groups has a high consumption of ultra-processed options instead of fresh foods. These findings may assist professionals when advising JDM patients about the importance of eating healthily.

The high prevalence of physical inactivity found in JDM patients has also been reported by other authors in regard to rheumatological pediatric diseases [8]. Some patients have physical limitations, joint involvement, and difficulties in accompanying their colleagues in their physical activities. The high prevalence of physical inactivity may also be explained by social distancing and stay-at-home orders issued in cities across the globe that resulted in the reduction of opportunities for physical activity among children, particularly those in urban areas living in small apartments [43]. Encouraging exercise is a safe and effective strategy to preserve lean mass and to improve overall health and quality of life in JDM patients and can be suggested as a nonpharmacologic adjuvant treatment [44,45].

The gold standard when evaluating body composition is the four-compartment model, which uses body weight or mass, total body volume, total body water and bone mineral. However, this method, and others, such as quantitative computed tomography and magnetic resonance imaging, generally have a limited usefulness for clinicians, since they are expensive, require highly specialized equipment and technicians and may expose children to radiation and/or the need for sedation [46]. Despite the limitations inherent to the age group, recent studies suggest that BIA-derived body composition and phase angle measurements are valuable when assessing nutritional status and growth in children [46]. To assess the nutritional status of children, regardless of their clinical condition, both fat and lean mass should be considered [11]. Previous studies have reported that BIA (Biodynamics 450) has a good predictive capacity to evaluate body composition in children [47] and adolescents [48]. BIA is based on the passage of an electric current of low intensity on the body, determining the values of impedance, resistance, reactance and PhA, through which body composition is estimated [11,13,46].

Another study performed by our study group in 2008 found lower lean mass in JDM, but no influence in fat mass [7]. Contrarily, our study did not find any difference between lean mass in patients and controls, but higher lean mass in patients with PhA ≥ 5.5. Lean mass is mostly constituted by body water, which is an excellent conductor of electricity and offers low resistance to the electric current. This means that low resistance values directly contribute to the increase of phase angle [10,46]. In a group of adolescents, PhA was directly associated with physical fitness indicators, showing that this parameter can be used to monitor the health of adolescents [49].

PhA is considered a marker of body cell mass as it reflects cell membrane integrity and is an important prognostic factor in many diseases [50]. A low phase angle score reflects an impaired cellular membrane integrity, suggesting cell death and breakdown in the selective permeability of cell membranes [11,49]. On the other hand, a larger PhA suggests a greater number of intact cell membranes, body cell mass, and healthy cell membranes [50]. PhA is a screening tool used to identify patients at risk of deterioration of nutritional status and functionality [11].

There is no published data regarding PhA in JDM patients. As mentioned by the American Society for Parenteral and Enteral Nutrition (ASPEN) in 2006, the use of bioelectrical impedance PhA has been recommended as a prognostic tool in the clinical setting, though published reference data are lacking for children [13]. The Third National Health and Nutrition Examination Survey (NHANES III) presented PhA data (8.0 ± 1.0 for males and 7.5 ± 0.8 for females) according to gender and ten-year age range of a representative sample of healthy American individuals (mean age 15.4 ± 2.3 for males and 15.3 ± 2.3 for females) [51]. Because PhA differs by age and gender, it becomes difficult to compare values across populations of different genders and age groups. Moreover, the Brazilian population is ethnically diverse, reinforcing the reason for not using NHANES values as cut-off points in this study.

As we found a positive correlation between PhA and disease duration (*p* < 0.001), additional comparisons were performed as exploratory analyses to confirm that data. These analyses were based on the median [52] PhA in JDM patients (5.5) in order to compare the extremes of our population. Due to the limitation of sample size, analyses based on the extreme percentiles were not possible. Lower PhA values have been associated with mortality in critically ill children, demonstrating that PhA can be a potential prognostic marker [12]. Additionally, lower PhA and other nutritional variables have been observed in juvenile idiopathic arthritis children compared with healthy peers, suggesting the use of PhA as an indicator of nutritional status as it can be useful in identifying risk of malnutrition [11].

A negative correlation between PhA and DAS total score, DAS muscle and DAS skin can be explained by the disease course. As it is a marker of muscle mass, cellular function and nutritional status, PhA may be a predictive factor for the risk of different complications [11]. Besides that, the shorter duration of the disease is related to the onset of a more severe disease and, therefore, with lower PhA values.

It is already known that body composition measurements in pediatric patients are inherently challenging, due to the rapid growth-related changes in height, weight, and lean and fat mass; however, they are fundamental for the quality of the clinical follow-up [53]. We found less than 10% of patients with lipodystrophy, with no relationship with bioelectrical impedance. The Slaughter equation was found to be a good estimate of the percentage of BF, similar to the results of electrical bioimpedance in this study.

A study performed with German children and adolescents found, for the general population, a PhA = 5.48 for girls and a PhA = 5.5 for boys, stratified for BMI and age. Arm muscle circumference, arm muscle area and lean mass higher in patients with PhA ≥ 5.5 can be associated with a better prognosis even with a longer disease duration, probably due to the protective effect related to improving health in general [13]. The higher values of DAS total score, DAS skin and DAS muscle in patients with PhA < 5.5 can be understood because a lower PhA is frequently associated with the disease and its influences, such as infection, inflammation or disease activity-specific parameters [10]. Disease activity scores in patients with PhA ≥ 5.5 were lower, showing the importance of initial aggressive treatment to enter remission [53,54].

The limitations of this study can be related to the cross-sectional model, in addition to the small number of patients and controls enrolled due to the rarity of JDM. Additionally, the inclusion of patients from one single reference center may preclude generalizations of conclusions to larger populations. The fact that data collection was also carried out during the COVID-19 pandemic may reflect weight gain and physical inactivity within an obesogenic environment for patients and controls. Furthermore, it would have been interesting to investigate the participant’s type and intensity of physical activity and other possible prognostic values in the JDM population. Additionally, it is known that dual energy X-ray is considered the gold standard method by which to evaluate body composition, although it requires costly technology. Therefore, electrical bioimpedance as emerged as an accurate and consistent tool that is noninvasive, portable, operationally simple and presents a feasible alternative.

## 5. Conclusions

We have demonstrated that bioelectrical impedance can serve as an auxiliary exam in the medical and nutritional follow-up of JDM patients, as it seems to impact functional ability. These findings may assist professionals when advising JDM patients about the importance of physical activity and of eating healthily in preserving lean mass.

## Figures and Tables

**Table 1:** Demographic, anthropometric, clinical, and therapeutical data and food intake in juvenile dermatomyositis patients and healthy controls.

Parameters	JDM Patients (n = 30)	Healthy Controls (n = 24)	*p*-Value	Effect Size
Demographic parameters				
Sex, male (%)	12/30 (40)	10/24 (41.7)	0.924	
Age (years)	11.8 ± 4.0	10.7 ± 3.1	0.249	
Anthropometric data/Nutritional status				
Weight (kg)	42.1 ± 15.9	38.9 ± 10.5	0.385	
Height (cm)	143.6 ± 17.9	142.7 ± 13.6	0.836	
Z-score Height/age	−0.46 ± 0.8	0.35 ± 0.7	<0.001	−1.08
Body mass index, BMI (kg/m^2^)	19.7 ± 4.2	18.8 ± 2.7	0.369	
Z-score BMI/age	0.45 ± 1.5	0.64 ± 1.1	0.627	
Malnutrition (%)	2 (6.7)	0 (0)	0.496	
Eutrophy (%)	17 (56.7)	16 (66.7)	0.576	
Overweight/obesity (%)	11 (36.7)	8 (33.3)	1.000	
Waist circumference/height > 0.5	11 (36.7)	6 (25)	0.469	
Physical inactivity				
“Once a week”, “rarely” and “never” (%)	21 (70)	13 (54.2)	0.324	
Body composition				
Body fat (kg)	11.6 ± 6.7	9.8 ± 3.9	0.216	
Body fat (BF) (%)	26.0 ± 8.2	24.5 ± 4.4	0.377	
BF Very low/low (%)	4 (13.3)	0 (0)	0.120	
BF Normal (%)	6 (20)	2 (8.3)	0.277	
BF High/very high (%)	20 (66.7)	22 (91.7)	0.046	
Lean mass (kg)	30.3 ± 10.3	29.1 ± 6.9	0.611	
Lean mass (%)	73.9 ± 8.2	75.5 ± 4.4	0.374	
Slaughter equation (BF%)	26.2 ± 8.2	27.9 ± 8.1	0.466	
Deurenberg equation (BF%)	21.5 ± 5.4	21.1 ± 4.1	0.760	
Basal metabolic rate (BMR) (kcal/day)	956.2 ± 321.1	907.9 ± 217	0.533	
Phase angle (PhA)	5.2 ± 1.3	6.1 ± 1.0	0.016	−0.74
Food intake				
Energy intake (EI) (Kcal/day)	1500.4 (1082.8–2723.9)	1593.7 ± 425.5	0.835	
Carbohydrate (g)	204.7 (127–398.3)	186.8 ± 60.7	0.091	
Carbohydrate (%EI)	55.5 ± 9.6	47.5 ± 11.9	0.014	0.74
Protein (g)	59.3 (30.5–163.4)	72.5 (39.2–179.7)	0.139	
Protein (%EI)	16.9 ± 6.4	20.5 ± 8.1	0.073	
Fat (g)	43.1 (17.5–110.7)	49.4 (27.6–123.1)	0.251	
Fat (%EI)	27.7 ± 7.1	32 ± 7.6	0.068	
Monounsaturated fat (g)	12.3 ± 9.8	14.5 ± 11.0	0.398	
Polyunsaturated fat (g)	6.7 ± 5	8.1 ± 8.7	0.931	
Saturated fat (g)	17.6 (2.9–56.1)	19.2 (7.6–36.5)	0.403	
Total Fiber (g)	17.3 (2.6–40.9)	11.9 (4.6–25.1)	0.000	0.90
Vitamin A (RE)	254.1 (0.0–988.5)	286.1 (0.0 –1335.1)	0.503	
Vitamin C (mg)	27.9 (0.9–1517.2)	43.1 (2.3–1455.3)	0.375	
Vitamin E (mg)	3.9 (0.9–221.5)	6.2 (0.7–25)	0.862	
Zinc (mg)	6.8 (2.1–32.3)	6.1 (2.5–17.7)	0.862	
Selenium (mcg)	28.2 (4.2–108.8)	49.4 (3–172.5)	0.144	
Calcium (mg)	372.9 (42.6–1264.3)	447.4 (53.4–1293.7)	0.508	
Health Related Quality of Life (HRQL) Scores				
CHAQ (0–3)	0.5 (0.0–1.4)	0.0 (0.0–0.6)	0.001	1.06
Peds Ql parents (0–100)	65.8 (9.8–100)	88.0 (47.8–100)	0.212	
Peds Ql patient (0–100)	66.8 ± 22.8	87.2 ± 13.6	0.001	−0.34

Results are presented in n (%), median (minimum and maximum values) or mean ± standard deviation. CHAQ—Childhood. Health Assessment Questionnaire; HRQL—Health Related Quality of Life; JDM—Juvenile Dermatomyositis; Peds Ql—Pediatric Quality of Life Inventor. Food intake: nutrients units of grams (g), milligrams (mg), micrograms (mcg), retinol (RE).

**Table 2:** Adequacy of dietary intake by juvenile dermatomyositis patients and healthy controls based on the Dietary Reference Intake (DRI).

Parameters	JDM Patients (n = 30)	Healthy Controls (n = 24)	*p*-Value
Carbohydrate (%EI)			
Within AMDR	22 (73.3)	16 (66.7)	0.765
Above AMDR	2 (6.7)	0 (0)	0.497
Protein (%EI)			
Within AMDR	27 (90)	18 (75)	0.165
Above AMDR	2 (6.7)	4 (16.7)	0.389
Total Fat (%EI)			
Within AMDR	13 (43.3)	12 (50)	0.784
Above AMDR	5 (16.7)	8 (33.3)	0.206
Saturated fat (%EI)			
Above AMDR	23 (76.7)	22 (91.7)	0.270
Total Fiber (g/day)			
Below recommendation	16 (53.3)	17 (70.8)	0.263
Vitamin A (RE)			
Within recommendation	7 (23.3)	4 (16.7)	0.736
>UL	1 (3.3)	0 (0)	>0.999
Vitamin C (mg)			
Within recommendation	12 (40)	11 (45.8)	0.784
>UL	1 (3.3)	2 (8.3)	0.579
Vitamin E (mg)			
Within recommendation	6 (20)	7 (29.2)	0.528
>UL	0 (0)	0 (0)	>0.999
Zinc (mg)			
Within recommendation	11 (36.7)	11 (45.8)	0.582
>UL	0 (0)	0 (0)	>0.999
Selenium (mcg)			
Within recommendation	9 (30)	15 (62.5)	0.027
>UL	0 (0)	0 (0)	>0.999
Calcium (mg)			
Within recommendation	3 (10)	3 (12.5)	>0.999
>UL	0 (0)	0 (0)	>0.999

Results are presented in n (%). EI—energy intake; AMDR—acceptable macronutrient distribution ranges; JDM—juvenile dermatomyositis; RE—retinol equivalent; UL—upper level. Intakes are on a per day basis.

**Table 3:** Demographic, anthropometric, clinical data and food intake in juvenile dermatomyositis patients according to phase angle.

Parameters	JDM Patients with PhA < 5.5	JDM Patients with PhA ≥ 5.5	*p*-Value	Effect Size
	(n = 14)	(n = 16)		
Demographic parameters				
Current age (years)	9.8 ± 3.1	14.1 ± 3.9	0.003	−1.19
Disease duration (years)	2.2 (0.2–8.6)	7.5 (2.7–16.6)	<0.001	1.04
Anthropometric data/nutritional status				
Weight (kg)	33.8 ± 11.0	49.2 ± 16.4	0.005	−1.09
Height (cm)	134.8 ± 15.8	151.2 ± 16.4	0.010	−1.01
Z-score height/age	−0.38 ± 0.9	−0.57 ± 0.7	0.547	
Body mass index, BMI (kg/m^2^)	18.2 ± 3.9	20.9 ± 4.1	0.074	
Z-score BMI/age	0.36 ± 2.0	0.47 ± 1.2	0.859	
Malnutrition (%)	2 (14.3)	0 (0)	0.209	
Eutrophy (%)	7 (50)	10 (62.5)	0.713	
Overweight/Obesity (%)	5 (35.7)	6 (37.5)	1.000	
Waist circumference (cm)	66.3 ± 9.8	74.7 ± 11.6	0.042	−0.77
Waist circumference/height	0.42 ± 0.27	0.49 ± 0.06	0.377	
Arm circumference (cm)	20.7 ± 4.2	24.4 ± 4.8	0.033	−0.79
Arm muscle circumference (cm)	15.6 ± 3.0	19.3 ± 4.1	0.010	−0.98
Arm muscle area (cm^2^)	20.7 (7.3–40.4)	27.1 (16.4–70.3)	0.017	−0.90
Body composition				
Body fat, BF (kg)	9.3 ± 5.5	13.7 ± 7.2	0.066	
Lean mass, LM (kg)	24.3 ± 6.1	35.5 ± 10.5	0.001	−1.28
Bioelectrical impedance (LM%)	74.3 ± 8.8	73.7 ± 7.9	0.851	
Bioelectrical impedance (BF%)	25.7 ± 8.8	26.3 ± 7.9	0.851	
Slaughter equation (BF%)	24.9 ± 8.6	27.4 ± 8.0	0.414	
Deurenberg equation (BF%)	20.5 ± 4.6	22.2 ± 6.1	0.382	
Basal metabolic rate, (BMR) (kcal/day)	768.4 ± 194.1	1108.7 ± 327.0	0.002	−1.23
Lipodystrophy (%)	1 (7.1)	1 (6.2)	1.000	
Physical inactivity				
“Once a week”, “rarely” or “never” (%)	9 (64.3)	12 (75)	0.694	
HRQL and JDM scores				
Peds QL parents 4.0 (0–100)	66.6 ± 20.2	67.1 ± 25.6	0.956	
Peds QL patients 4.0 (0–100)	76.4 ± 17.0	75.4 ± 13.9	0.863	
CHAQ (0–3)	0.68 ± 0.47	0.35 ± 0.44	0.061	
DAS total score	2 (0–15)	0.5 (0–3)	0.015	0.52
DAS skin	1 (0–7)	0.5 (0–3)	0.031	0.35
DAS muscle	1 (0–8)	0 (0–0)	0.014	0.57
Cumulative drug therapy				
Corticosteroid (g)	9.1 ± 8.3	14.9 ± 11.0	0.110	
Duration of prednisone use (days)	554.5 (0–1210)	842.5 (0–3358)	0.092	
Methotrexate, MTX (g)	1.5 (0.1–7.8)	3.2 (0–13.3)	0.168	
Duration of MTX use (days)	731.5 (56–1210)	1105.5 (0–3891)	0.058	
Azathioprine (g)	0 (0–41.4)	0 (0–184.8)	0.188	
Duration of azathioprine use (days)	0 (0–528)	0 (0–1762)	0.188	

Results are presented in n (%), median (minimum and maximum values) or mean ± standard deviation. CHAQ—Childhood Health Assessment Questionnaire; DAS—Disease Activity Score; JDM—juvenile dermatomyositis; HRQL—health-related quality of life; Peds Ql—Pediatric Quality of Life Inventory; PhA—Phase angle.

## Data Availability

The individual data concerning the participants are confidential due in order to secure its privacy according to the ethics committee. If necessary, contact the corresponding author.
